# Using light to improve commercial value

**DOI:** 10.1038/s41438-018-0049-7

**Published:** 2018-09-01

**Authors:** Matthew Alan Jones

**Affiliations:** School of Biological Sciences, University of Essex, Wivenhoe Park, Essex, Colchester, CO4 3SQ UK

## Abstract

The plasticity of plant morphology has evolved to maximize reproductive fitness in response to prevailing environmental conditions. Leaf architecture elaborates to maximize light harvesting, while the transition to flowering can either be accelerated or delayed to improve an individual’s fitness. One of the most important environmental signals is light, with plants using light for both photosynthesis and as an environmental signal. Plants perceive different wavelengths of light using distinct photoreceptors. Recent advances in LED technology now enable light quality to be manipulated at a commercial scale, and as such opportunities now exist to take advantage of plants’ developmental plasticity to enhance crop yield and quality through precise manipulation of a crops’ lighting regime. This review will discuss how plants perceive and respond to light, and consider how these specific signaling pathways can be manipulated to improve crop yield and quality.

## Tailoring the perfect crop with light

Crop yields and qualities can be improved by manipulating their photoreceptors with light. Matthew Jones of the UK’s University of Essex reviewed the latest research on plant responses to various wavelengths of light and how photoreceptor pathways can be manipulated to improve commercial crops. Plants contain a variety of photoreceptors, each responding to distinct wavelengths of light to initiate cellular pathways that affect plant growth and development. These pathways can lead to the formation of pigments that improve fruit quality. They also play roles in plant responses to shade, daily and seasonal changes in light, and flowering times. Recent developments in cost-effective LED lights can lead to the optimization of crop growth. However, further research is needed to understand the differences in photoreceptors and their pathways in various plants.

## Introduction

The effective application of light is essential for plant husbandry, but the demands of modern, intensive horticulture often conflict with the optimal planting strategy for plant growth. Dense planting regimes induce shading throughout the canopy, with individual plants striving to optimize light harvesting at the expense of their neighbors. This intra-crop competition leads to a varied light environment that has consequences for crop uniformity and total yield, which is exacerbated by changing light availability over the course of the year^1^. Historically, horticulturalists have sought to mitigate these effects through the development of varieties with altered developmental responses that improve harvest. Alternatively, enclosed glasshouses enable control of light, temperature, humidity, and CO_2_, each of which can alter plant development. The recent advent of commercially-viable LED-based lighting provides an additional opportunity to optimize plant development through the application of specific light wavelengths at times most appropriate to optimize crop traits. These manipulations will be of immediate benefit for glasshouse-grown plants where supplemental light can be readily provided, although as LED technology advances there will be opportunities to apply similar approaches in the field. This review will summarize our understanding of plant perception and photomorphology and how this can be applied to optimize plant growth.

## Plant photoreceptors

As photosynthetic organisms, plants need to harvest sufficient light energy to sustain growth and reproduce. However, it is not sufficient to simply irradiate plants with a single quality of light. Although monochromatic red or blue light sources (as chlorophyll predominantly absorbs light in the red and blue portions of the spectrum) can be used to cultivate crops, such plants develop atypically. This is likely because of the imbalanced activation of different photoreceptors which ultimately impairs photosynthesis either through inappropriate stomatal behavior or incorrect accumulation of photosynthetic pigments^2,3^. Plants sense light both through specific photoreceptors as well as by monitoring the metabolic consequences of photosynthesis^4,5^, thereby allowing  light to be used as a predictive environmental indicator as well as an energy source. Shortening days imply the onset of winter and subsequent reductions in temperature whilst the spectrum of light provided by the sun is enriched in the blue portion of the spectrum at dawn and dusk relative to midday^6^. Given these environmental characteristics, plants have evolved sophisticated mechanisms to determine light availability and quality. Decades of research have revealed a complex network of photosensory pathways that enable plants to precisely respond to light quantity, quality, and duration^5,6^. Perhaps more importantly, plants are able to respond and adapt to each of these stimuli. In an evolutionary context, plants responses to light have been selected to maximise their survival; the challenge facing horticulturalists is how these existing light-responsive traits can be modified or selectively activated to increase yield and crop quality.

**Fig. 1:**
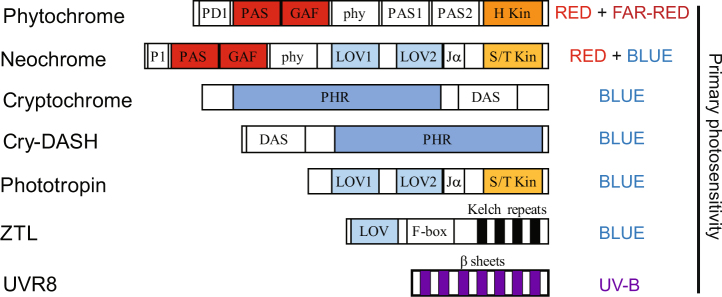
Schematic diagram illustrating major domain structure of plant photoreceptors. Domains necessary for red light detection are shown in red, whilst those for blue light detection are shown in blue. The N-terminal phytochrome PAS and GAF domains interlink to allow binding of a phytochromobilin chromophore whilst the cryptochrome PHR domain associates with FAD and MTHF chromophores. LOV domains bind a FMN chromophore. Kinase domains are highlighted in orange. *DAS* ***D****rosophila*, ***A****rabidopsis*, ***S****ynechocystis* cryptochrome domain, *FAD* **F**lavin **A**denosine **D**inulceotide, *FMN* **F**lavin **M**ono-**N**ucleotide, *GAF* *c****G****MP* specific and -regulated cyclic nucleotide phosphodiesterase, **A**denylyl cyclase, and **F**hlA, *H Kin* **H**istidine **kin**ase, *Jα* Jα-helix, *LOV* **L**ight/**O**xygen/**V**oltage sensitive, *MTHF* **M**ethenyl**t**etra**h**ydro**f**olate, *PAS* **P**er/**A**rnt/**S**im, *PD1* **P**hytochrome **D**omain 1, *PHR* **P**hotolyase **H**omology **R**egion, phy-**Phy**tochrome domain 4, *S/T Kin* **S**erine/**T**hreonine **kin**ase

In contrast to animals, which have evolved specialized light sensing organs, plants perceive light in a cell-autonomous fashion. Plants have evolved a suite of photoreceptors (Fig. 1), each of which provide sensitivity to different portions of the light spectrum by binding a light absorbing co-factor (referred to as a chromophore^7^). Red and far-red light (600–750 nm) is primarily detected by the phytochrome family^8^ while blue and UV-A light (320–500 nm) is sensed by cryptochromes, phototropins, and members of ZEITLUPE/ADAGIO family^7,9–11^. UV-B light (290–320 nm) is perceived by the UVR8 photoreceptor^12^. In addition to these characterised photosensors, plants are also able to respond to ‘green’ light (500–600 nm), although the photoreceptors responsible for these responses have not been elucidated^13^. The existence of distinct photoreceptor families provides opportunities to selectively activate individual pathways, thereby precisely controlling plant development.

### Phytochromes

Phytochromes were initially identified in 1959 as the photoreceptor that mediates plant photomorphogenesis in response to long-wavelength visible light^14^. The phytochrome family has since been found to be ubiquitous amongst seed plants and cryptophytes, with examples also being found in cyanobacteria, non-photosynthetic bacteria, and fungi^15^. Phytochromes (phy) are sensitive to irradiation by both red and far-red light, and uniquely function by measuring the relative quantity of each of these wavelengths^15^. The phytochrome basal state (designated P_r_) is sensitive to red light and upon irradiation is converted to a far-red sensitive state (P_fr_). Reversion to the P_r_ form occurs either after far-red light exposure or as a consequence of dark incubation. The relative amounts of each of these forms determine downstream signalling events, with the P_fr_ form considered to be the active signalling state^16^.

**Fig. 2:**
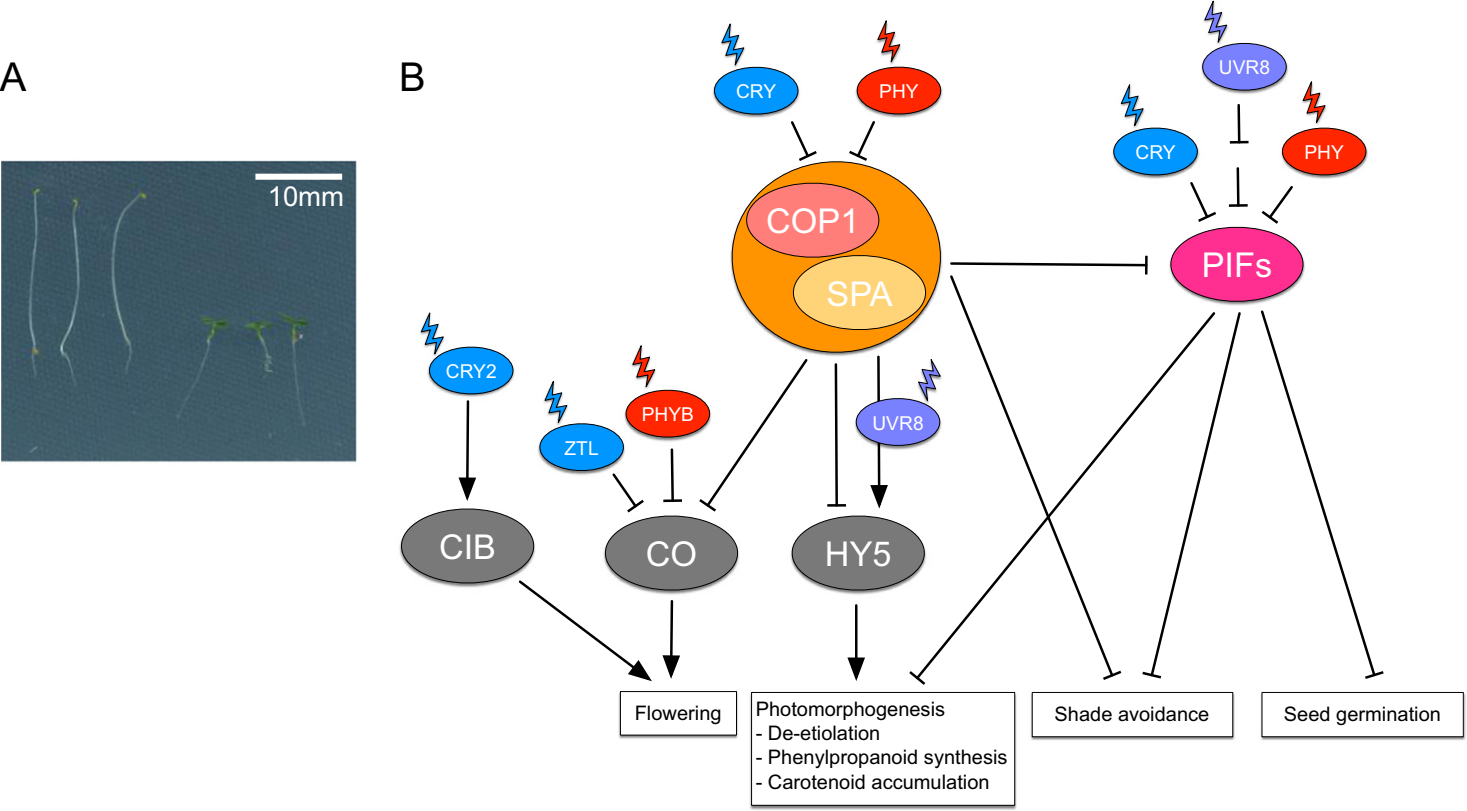
Photomorphogenesis is regulated by conserved signalling hubs. **a** In the absence of light, seedlings have an etiolated phenotype (left). Upon perceiving light, plants initiate photomorphogensis leading to dramatic changes in plant architecture including cotyledon expansion and the inhibition of hypocotyl elongation (right). **b** Cryptochromes, phytochromes, and UVR8 perceive blue, red, and UV-B light respectively (see the section 'Plant photoreceptors'). Phytochromes and cryptochromes inhibit the activity of both the COP1/SPA and PIF signalling hubs, leading to changes in gene expression that culminate in photomorphogenesis and shade avoidance responses. Activated UVR8 modulates the function of the COP1/SPA complex to promote photomorphogenesis. The COP1/SPA complex has additional roles in the regulation of flowering, while PIFs influence seed germination. Cryptochromes and phytochromes also influence plant development independently of these signalling hubs; for instance CRY2 (see the section 'Cryptochromes') accelerates flowering via CIB transcription factors whereas phyB (see the section 'Phytochromes') inhibits CO accumulation in the morning independently of COP1 (see the section 'Shade avoidance'). *CIB* CRYPTOCHROME INTERACTING BASIC HELIX LOOP HELIX, *CO* CONSTANS, *COP1* CONSTITUTIVELY PHOTOMORPHOGENIC1, *CRY* Cryptochrome, *HY5* ELONGATED HYPOCOTYL5, *PHY* Phytochrome, *PIF* PHYTOCHROME INTERACTING FACTOR, *UVR8* UV-B RESISTANCE LOCUS8, *ZTL* ZEITLUPE

Higher plant genomes encode a suite of phytochrome proteins, each with slightly diverged light-sensitivity and function. Angiosperm phytochromes can be placed into two broad groups based upon the stability of the red light irradiated P_fr_ form. Type I phytochromes (such as phyA) accumulate in the dark and are rapidly degraded after illumination^17^. Type I phytochromes are primarily involved in very low light responses (VLFR) or those involving a high irradiance response (HIR), two signalling modes that are functionally different and appear to operate through at least partially distinct pathways^18^. Type II phytochromes (such as phyB-E) remain stable in the presence of light allowing these phytochromes to respond persistently to fluctuations in illumination (low fluence response, LFR^19,20^. LFR responses (such as shade avoidance) are reversible and are determined by the ratio of red and far red light used to irradiate the plant^21^. VLFR, HIR, and LFR interact to facilitate light sensitivity under a broad range of light conditions. As phyA is light-labile, phyA is generally considered to be the primary photoreceptor in etiolated seedlings, with phyB and other type II phytochromes having greater importance in light-grown plants with regards shade avoidance and the regulation of flowering time (Fig. 2^21^).

### Cryptochromes

Plant cryptochromes are blue light photoreceptors that are one of five subfamilies identified in the photolyase/cryptochrome family based on molecular phylogenetic analyses and functional similarity^22^. Cryptochromes have been identified in the model plant *Arabidopsis thaliana*, the closely related *Brassica napus*, and in a number of other model plant systems including pea, rice, and tomato^10^. The majority of plant genomes studied encode for two canonical plant cryptochrome proteins (CRY1 and CRY2) and one member of the CRY-DASH subfamily, which has been designated CRY3 (Fig. 1^23–26^).

Cryptochromes perceive blue light via a flavin adenine dinucleotide chromophore, with blue light irradiation triggering conformational changes that culminate in cryptochrome dimerization and the activation of biochemical signalling pathways^9,27^. While CRY1 is stable when illuminated, CRY2 is degraded after light activation^25,28,29^. Cryptochromes largely induce changes in plant development through changes in gene expression^30,31^. These changes in gene expression induce physiological changes from de-etiolation through to flowering, and also have a role in the production of anthocyanins (Fig. 2^32^). Cryptochromes have been found associated with DNA, but also activate CRYPTOCHROME INTERACTING BASIC HELIX LOOP HELIX (CIB) transcription factors and the CONSTITUTIVELY PHOTOMORPHOGENIC1 (COP1) and PHYTOCHROME INTERACTING FACTOR (PIF) signalling hubs (Fig. 2^33,34^).

### Phototropins

Phototropins are plasma membrane-localised protein kinases which were initially characterised in *Pisum sativum* membrane extracts due to their blue-light-dependent phosphorylation^35^, Fig. 1). As in other photoreceptors, blue light induces conformational changes that generate a biologically-active state which gradually reverts to the dark-adapted form in the absence of light^9^. Since the identification of the *PHOT1* locus in *Arabidopsis*^36^, phototropins have been characterised in numerous other dicots and monocots, as well as in lower plants such as the fern *Adiantum capillis-veneris*^37^. Studies have identified two primary members of the phototropin family, phototropin (phot) 1 and 2^36,38,39^, both of which are found in *Arabidopsis*. The phots have partially redundant roles in many responses in *Arabidopsis*, but have some diverged functions; in general phot1 is sensitive to lower fluences of light while phot2 acts in response to higher light intensities^40^. Like phytochromes and cryptochromes, phots are capable of eliciting changes in gene expression in response to blue light stimulation, although compared to the modulation of gene expression induced by cryptochrome activity this role is minor^41^. Instead, phots are thought to act primarily at a post-transcriptional level to mediate responses to blue light. Phototropins have been shown to be the primary light receptors for a range of blue light-specific responses including phototropism (after which they were named), chloroplast accumulation, leaf positioning and expansion and also stomatal opening^42^. In addition, phot2 induces chloroplast avoidance movements under high light irradiation^42^.

Phot1 and phot2 appear to have evolved from a single gene duplication event after the evolution of seed plants^36,39,43^. Single copies of *PHOT* are found in pteridophytes and in the single-celled algae *Chlamydomonas reinhardtii*^44,45^ and are likely derived from the ancestral *PHOT* gene^43^. In addition to these sequences, a chimeric photoreceptor (neochrome 1, neo1) has been identified in *Adiantum* and the alga *Mougeotia scalaris* which contains the red light-sensing N-terminal region of a phytochrome fused with a complete phototropin protein^46^. This fusion event allows both red and blue light to be used to induce what are primarily thought to be blue light-mediated phot-dependent responses in higher plants. This is thought to be advantageous in the shaded, low light environments in which these plants are commonly found^47^. Indeed, neochrome is thought to have arisen on two independent occasions in cryptophytes^46^.

### ZEITLUPE family

The ZEITLUPE (ZTL) family consists of three members; ZEITLUPE (ZTL), FLAVIN BINDING, KELCH REPEAT, F-BOX1 (FKF1) and LOV KELCH PROTEIN2 (LKP2)^48–50^. Each of these proteins have a conserved structure consisting of an N-terminal LOV domain, an F-box domain which allows binding to a SKP1–CUL1–FBP (SCF) ubiquitin ligase, and a region of kelch repeats which are also thought to allow protein–protein interactions^51^. The existence of a light sensitive LOV domain coupled with an F-box suggested that these proteins may be involved in the light-dependent regulation of protein stability. Indeed, recent work has shown a role for ZTL and FKF1 in the circadian system where their light-dependent function allows modulation of internal timing signals^52–54^. This mechanism allows plants to induce flowering at favorable times of year by responding to seasonal changes in day length through light-dependent modulation of circadian clock signals^52,55^ (see the section 'Photoreceptors contribute to temperature sensitivity and endogenous timing signals').

### UVR8

Although not detected by the human eye, sunlight contains a small proportion (<0.5%) of UV-A (315–400 nm) and UV-B (280–315 nm) light^56^. Plants perceive light via the UV-B RESISTANCE8 (UVR8) photoreceptor^57,58^, with loss of this photoreceptor leading to enhanced susceptibility to UV-B radiation^59^. UVR8 disassociates from its homodimer in the presence of UV-B light, with the resultant monomers binding with partners such as COP1 to induce changes in gene expression^60–63^. Although damaging in large quantities, UV-B induced signalling via the UVR8 pathway also has important benefits, promoting pest resistance, increasing flavonoid accumulation in fruits, improving photosynthetic efficiency, and serving as an indicator of direct sunlight and sunflecks^56,64–68^.

## Photoreceptors contribute to temperature sensitivity and endogenous timing signals

### Activated photoreceptors contribute to temperature perception

Although light serves a vital role in plant development it is important to consider how photoperception is integrated with other environmental information such as ambient temperature and time of day. Although a thorough discussion of plants responses to temperature are outside the scope of this review (see^69,70^ for recent overviews) it is becoming apparent that photoreceptors directly contribute to temperature perception. Recent work reveals that the stability of the light-activated states of phytochromes and phototropins is prolonged at lower temperatures through retardation of dark reversion^71–73^. This modulation of light signalling pathways by temperature allows immediate integration of these important environmental signals. This is particularly important in the context of LED lighting systems where the utilization of monochromatic light sources may have unintended consequences for plants perception of temperature through the specific activation of individual families of photoreceptors.

### Plants responses to light are informed by the circadian system

While we have characterized many of the photoreceptors utilized by plants (see the section 'Plant photoreceptors') it is also apparent that biological timing mechanisms have arisen that regulate plants’ responses to these signals^4,74^. The circadian system is an internal timekeeping mechanism that consists of interlocking transcription/translation loops that generate an approximate 24-h cycle^75^. Approximately one third of the expressed transcriptome is regulated by the circadian system, with transcription of phytochromes, cryptochromes, phototropins, and UVR8 each being regulated by the circadian system^76–78^. In addition, the clock modulates photosensory pathways such that plants perception of light also varies during the day, a concept known as circadian gating^74,79^. The biological clock allows plants to anticipate daily environmental changes as well as acting as a reference to measure seasonal changes in day length^75,80^, consequently contributing to flowering time in photoperiod-sensitive species (see the section 'Photoperiodic control of flowering time').

Conversely, the circadian system is highly responsive to light, a quality necessary to ensure accurate perception of changing day lengths during the year. The loss of cryptochromes, or the removal of individual or multiple phytochromes, alters the progression of the circadian cycle under constant blue or red light respectively^81–83^. The ZTL family of blue light photoreceptors, named after the predominant member ZEITLUPE (ZTL), have similarly been shown to have a role in regulating the circadian system, with the other two ZTL family members, LKP2 and FKF1, providing partial redundancy for ZTL function^84,85^. The temporal regulation initiated by the clock, and its sensitivity to light, provide additional opportunities to precisely control crop development in response to light and should be considered when designing optimal lighting regimes for crops.

## Plant development is controlled by light

Light is perhaps the most important consideration for optimizing plant growth, with light being utilized as both an energy source and as a developmental signal. All aspects of plant development are responsive to light, from germination through to the transition to flowering and fruit ripening^86^. The process by which developmental alterations occur in response to the changing light environment is referred to as photomorphogenesis^6^. In the absence of light newly-germinated seedlings have an etiolated phenotype with an extended hypocotyl (primary stem), an apical hook, and unopened cotyledons (embryonic leaves, Fig. 2a)^86^. These traits enable the seedling to rapidly emerge from the soil into the light at which point de-etiolation occurs, with dramatic consequences for seedling morphology. Light induces cotyledon expansion and the development of chloroplasts, thereby enabling photosynthesis, while hypocotyl elongation is curtailed. While this is perhaps the most dramatic light-induced developmental transition, light continues to be monitored throughout vegetative growth. Light intensity, duration, and spectral quality influence a range of vegetative characteristics including branching, internode elongation, leaf expansion, and orientation, with each of the photoreceptor families contributing via the photosensory network^6,87^. Light is also a fundamental signal necessary for the transition to flowering^6^, while the effects of light upon fruit development are also beginning to emerge.

Following photoperception phytochromes, cryptochromes, and UVR8, induce photomorphogenesis by inducing comprehensive changes in gene expression^30,88^. Much of plant photomorphogenesis is regulated via conserved modules, which are named after the originally identified components (Fig. 2). In the first module, COP1 acts with SUPPRESSOR OF PHYA (SPA) proteins to degrade a positive regulator HY5 in the dark^89–91^. In the presence of red or blue light, the COP1/SPA complex is inactivated by phytochromes and cryptochromes^89,92^, leading to the accumulation of HY5 and the induction of photomorphogenesis. Interestingly, UVR8 promotes photomorphogenesis through an alternative mechanism whereby UV-B activated UVR8 monomers associate with the COP1/SPA complex to promote HY5 accumulation^93^. The COP1/SPA complex also degrades CONSTANS, an essential component of the photoperiodic flowering pathway (see the section 'Photoperiodic control of flowering time'), and PIFs^94^. PIFs form the second regulatory hub^94^ and are also directly bound and inactivated by both phytochromes and cryptochromes; UVR8 indirectly inhibits PIF accumulation by repressing *PIF* transcription^95–101^. PIFs have important roles in regulating genes necessary for photomorphogenesis, but are rapidly degraded in the presence of light^94^. In addition, the light-induced degradation of PIFs can be limited by far-red light, thereby allowing PIFs to direct aspects of the shade avoidance response^102,103^. In combination, the COP1 and PIF signalling hubs integrate environmental information to control gene expression^89,94^.

### Light-induced pigments

#### Phenylpropanoids

Fruit quality is typically dependent upon the health of the bearing plants, although direct light irradiation also alters their biochemical composition^66^. One of the principle determinants of fruit quality is the accumulation of phenylpropanoids (including flavonols, anthocyanins, and proanthocyanidins), which alter the color, aroma, astringency, and antioxidant properties of fruit^104^. Importantly, light can have dramatic effects upon the quantity and types of flavonoids that accumulate (reviewed by^66^), although it should be noted that centuries of selective breeding have altered the specific responses of our crops (for example red vs. green apples^105^).

**Fig. 3:**
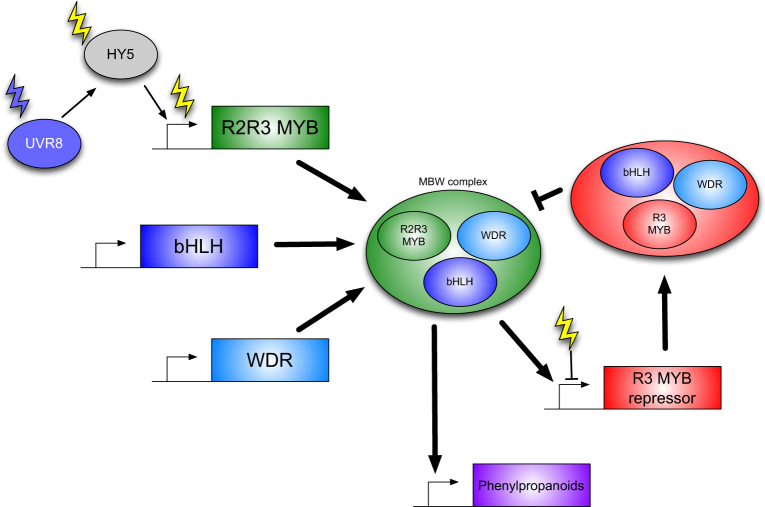
Phenylpropanoids accumulation can be induced by light. Phenylpropanoid accumulation is regulated by a conserved regulatory module comprising a R2R3 MYB, a bHLH, and a WDR transcription factor. Together these three proteins comprise the MBW complex that activates transcription of enzymes necessary for phenylpropanoid production. Of these three proteins, developmental and environmental induction of R2R3 MYBs is regulated to control MBW activity, in part via the transcription factor HY5. UVR8 stabilizes HY5 through modulation of the COP1/SPA complex, while other photoreceptors promote HY5 stability indirectly or act independently of HY5 (See Fig. 2). Additional control commonly occurs via feedback loops including closely related R3 MYBs that serve to repress MBW activity. R3 MYB transcription can be regulated by the MBW itself, or be independently repressed by light or other environmental and developmental signals. Genes are represented by rectangles, proteins by ovals. Green complexes activate gene expression, red components repress MBW activity. *bHLH* basic HELIX LOOP HELIX, *HY5* ELONGATED HYPOCOTYL5, *MBW* MYB/bHLH/WDR complex, *UVR8* UVB RESISTANCE LOCUS8

The spatial and temporal induction of phenylpropanoid metabolism occurs both post-transcriptionally and post-translationally via a conserved agglomeration of R2R3 MYB, bHLH, and WDR transcription factors known as the MBW complex (Fig. 3^66,106–109^). Regulation of the MBW complex by light subsequently leads to the altered accumulation of phenylpropanoids, although additional R3 MYBs are also capable of binding to the MBW complex to limit its activity^110^. For example, the R2R3 MYB transcription factor PAP1 is degraded by the COP1/SPA complex in the dark, leading to reduced anthocyanin accumulation (Figs. 2 and 3^111^), while UV-B light (via UVR8) induces transcription of R2R3 MYBs that induce flavonol accumulation in Arabidopsis and grape^112,113^. Interestingly, accumulation of phenylpropanoids can be increased by manipulating photoreceptor abundance in transgenic tomato and strawberry fruits, suggesting that activation of these photoreceptors using specific wavelengths of light could improve the nutritional value of fruits^114,115^.

#### Carotenoids

In addition to the regulation of phenylpropanoids, light also regulates the production of carotenoids as part of photomorphogenesis^116,117^. While carotenoids play a vital role in photosynthesis as part of the light harvesting complex^118^, they have also been adopted as photoprotectants, and have additional roles in growth and development^118^. In horticulture, carotenoids are valued as a valuable source of anti-oxidants and essential dietary precursors that accumulate in fruits and vegetables as they ripen^118,119^.

Light has been observed to affect carotenoid biosynthesis in a number of species during fruit ripening and flower development^120,121^. The carotenoid biosynthetic pathway is complex, and thoroughly reviewed elsewhere^118^. It is important to note, however, that one of the rate-limiting enzymes necessary for carotenoid biosynthesis, PHYTOENE SYNTHASE (PSY), is regulated by light. PSY activity is reversibly induced by red light, suggesting a role for phytochromes in this response^122^. It is likely that this regulation acts via COP1 (Fig. 2), as transgenic tomato fruits with reduced *COP1* or *HY5* transcript accumulation had altered carotenoid content^123^, although light induction of *PSY* transcript has also been reported in some species^124^. Encouragingly, studies using transgenic tomato to over-express phytochromes and cryptochromes observed increased carotenoid accumulation in transgenic fruits^114,125^, suggesting that enhancement of photoreceptor signalling could be sufficient to induce carotenoid accumulation.

### Shade avoidance

Modern horticulture requires plants to be grown in close proximity so as to generate a commercially-viable harvest, inevitably inducing a shade avoidance response as plants seek to outcompete their neighbors. Importantly, plants perceive and respond to changes in light quality before they are shaded, ensuring that most crops are responding to shade even if direct shading is avoided^102,126^. Plants absorb light in a wavelength-dependent manner, absorbing light in the UV and photosynthetically active portions of the spectrum (although comparatively less green) while reflecting far-red and infra-red light. As a consequence, plants are able to perceive shade as a change in either the quality or quantity of light^102,127,128^. Given phytochromes’ sensitivity to red/far-red light (see the section 'Phytochromes'), much research regarding shade avoidance (and consequently our understanding) concerns the role of these photoreceptors in mediating this response^102,126^. It is, however, important to note the role of blue, green, and UV portions of the spectra in governing plants responses to shade^63,98,128^.

Shade avoidance has many consequences for plant growth, ranging from leaf hyponasty (leaf movement), stem or petiole elongation, and directional growth away from shade of actively growing tissues, through to architectural changes such as reduced branching and increased leaf senescence that reduces resources devoted to shaded leaves^102,129,130^. These developmental changes ensure that plants are able to exploit any gaps in the canopy while also promoting vertical growth to over-shadow neighboring plants. Such developmental changes can also culminate in an acceleration to flowering in some species, with inactivation of phytochromes by far-red enriched light relieving repression of photoperiodic flowering (see the section 'Photoperiodic control of flowering time', ^131–133^). In commercial applications, such behavioral changes can potentially culminate in reduced yield, or in increased crop management (e.g., pruning) to minimize these consequences^134,135^, although such effects can be mitigated through the choice of alternate varieties.

### Photoperiodic control of flowering time

As part of the maturation process, plants undergo a transition to flowering that is largely irreversible^136^. The floral transition is consequently tightly regulated, with plants integrating day-length, age, and temperature cues to determine flowering time. These pathways combine to control the accumulation of FLOWERING LOCUS T (FT), which is the florigen transported from the leaves to the shoot apical meristem to initiate the floral transition in numerous species^137,138^. Given the importance of flowering to agriculture and horticulture, considerable time has been spent elucidating the molecular pathways underlying this control, although only light-induced pathways are considered here^80^.

**Fig. 4:**
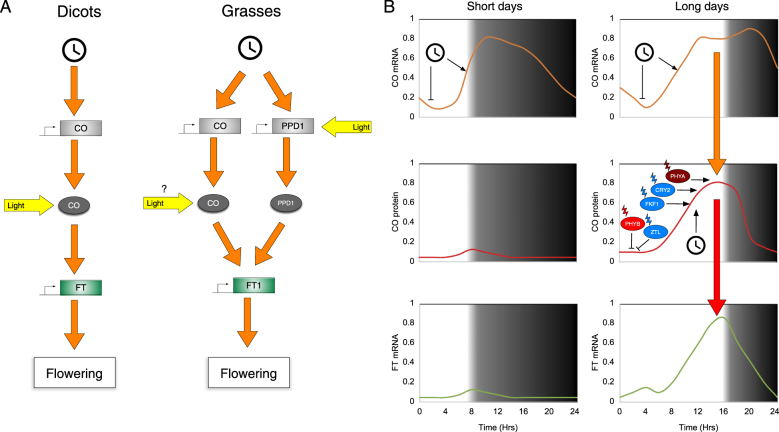
The floral transition is regulated by light. **a** Molecular control of photoperiodic flowering has arisen multiple times during evolution, but commonly requires circadian control of *CONSTANS* (*CO*) transcription. Post-translational stabilization of CO enables the transcription of *FLOWERING LOCUS T* (*FT*), which induces the floral transition in the meristem. An additional pathway has been described in grasses, where *PHOTOPERIOD1* (*PPD1*) transcription is induced by light and the clock. Both PPD1 and CO activate *FT* transcription in these species. **b** The external coincidence model explains how long day plants flower under inductive conditions. *CO* transcript (orange line, top) accumulates during the evening, but CO protein (red line, middle) only accumulates in the presence of light, when photoreceptors are necessary to inhibit CO degradation by COP1. Stabilization of CO protein in long days enables transcription of *FT*, culminating in floral transition. See also see the section 'Photoperiodic control of flowering time' and Fig. 2. Boxes indicate transcriptional targets, ovals represent protein. *CO* CONSTANS, *CRY* cryptochrome, *FKF1* FLAVIN BINDING KELCH REPEAT F-BOX1, *FT* FLOWERING LOCUS T, *PPD1* PHOTOPERIOD1, *PHY* phytochrome, *ZTL* ZEITLUPE

Flowering time in response to day-length is explained by the external co-incidence model, which is conserved across a wide-range of species (Fig. 4^80^). Transcription of a transcriptional activator, CONSTANS (CO), is controlled by the circadian system so that the protein accumulates during the late afternoon^80,137,139^. In particular, CYCLING DOF FACTORs (CDFs) prevent transcription of CO, but are degraded via a blue light-dependent pathway mediated by FKF1 in long days, allowing CO to accumulate under inductive conditions^52^. Importantly, CO protein is stabilized by blue or far-red light, with additional control mediated by clock-regulated factors^140–142^. This light-dependent regulation ensures that CO only accumulates in long days, and so *FT* transcription is limited to these permissive conditions in long day plants. Interestingly, red light limits CO accumulation in the morning^140,143,144^ suggesting that flowering may be suppressed in the absence of shade. Although Arabidopsis CO arose from a duplication during the divergence of the *Brassicaceae*, numerous examples indicate that regulation of FT by CO orthologues is a common consequence of convergent evolution^145–147^. For instance, a *CO* orthologue, Hd1, has been co-opted as a floral repressor in rice, a short day species^148^.

Additional photoperiodic flowering pathways have been identified in grasses such as barley and wheat (Fig. 4a). In these species *PHOTOPERIOD 1* (*PPD1*), a gene that arose from a duplication of a circadian clock gene after the divergence of the grasses, is important to integrate circadian and photoperiod information^149–151^. *PPD1* is expressed in the light via phytochrome C (phyC), and subsequently acts to promote expression of the *FT* homologue *FLOWERING LOCUS T1* (*FT1*)^151–153^. This pathway appears to act in addition to the CONSTANS-mediated pathway, although the relationship between CO-derived and PPD1-derived pathways remains to be fully tested^139^. It remains to be determined whether pathways analogous to PPD1 have arisen outwith the grasses.

## Improving crop yield using light

As light is a prerequisite for photosynthesis (and consequently plant growth) supplemental lighting is typically used to accelerate plant development^154–156^. Growers face many challenges in providing optimal lighting, with shade, cloud cover, and changing seasons introducing heterogeneity in both the spatial and temporal distribution of light. Given the broad range of light qualities perceived by plants it is apparent that at least one source of broad spectrum light should be provided (either from natural illumination, metal halide (MH), and High Pressure Sodium (HPS) lights, or from white or multi-spectral LED arrays). Beyond this requirement, many opportunities exist to manipulate the precise light environment used for plant growth to stimulate desirable plant development (such as fruit quality or delaying flowering to promote vegetative growth).

Supplemental overhead lighting has been used in glasshouses for many years to increase crop production during periods of low natural light, either to extend shorter winter days or during periods of inclement weather^154,156^. In general, a 1% increase in lighting provides a 1% increase in yield, although interactions between light and other factors (such as temperature and CO_2_) complicate this relationship^157^. Despite these obvious opportunities, numerous studies emphasize the varied responses of different crops to supplemental lighting regimes. It is also important to note that periods of darkness are often required to prevent chlorosis or impaired leaf development^158–162^. As a consequence it will be important to develop light regimes optimized for specific crops, with consideration of the local natural lighting environment, rather than applying a uniform lighting regime.

### Supplemental lighting and spectral manipulation

The development of LEDs that are cost effective to install at commercial scales exponentially increases the options available to growers as they seek to improve crop yield, with the opportunity to specify the quality, quantity, uniformity, and duration of light used^163^. LEDs also irradiate much less heat that their metal halide (MH) and high pressure sodium (HPS) predecessors, enabling novel strategies such as intra-canopy lighting to provide more uniform light throughout the canopy. Numerous studies demonstrate the utility of supplemental lighting, with improvements in crops ranging from lettuce leaves to the fruits of strawberries, cucumbers, sweet peppers, and tomatoes^164–167^. For instance, illumination of peppers with light was sufficient to induce color break, greatly improving commercial value^168^, while altering the ratio of blue and red light used to irradiate lambs lettuce (*Valerianella locusta*) improved yield and both sugar and phenol content of harvested leaves^165^. The individual sensitivities of plant photoreceptor families enables plant growth and development to be precisely controlled by changing the proportion of red/far-red/blue/UV LEDs used, with these light conditions changing plant architecture and flowering via pathways summarized in the ̔Plant development is controlled by light ʼ section. In future it will be necessary to refine our understanding of photoreceptor function in crops so that light regimes (including the precise light spectra used) can be optimized to improve yield and quality.

### Photoperiod extension

Perhaps the simplest utilization of supplemental lighting is to extend day length during the winter months. In some day neutral species, such as sweet peppers, day length extension photoperiod increased fruit yield, although comparable increases were not observed in closely related Solanaceae, such as tomatoes^160^. Interestingly, light quality has a profound effect on plant growth. For instance, the use of blue LEDs at the end of day improve tomato quality (although not yield^169^). As a consequence, it will be of great benefit to understand how photoreceptors contribute to these yield and quality phenotypes. Such knowledge will enable more a systematic approach to specifying light regimes for specific crops. This specification will depend upon both the local light environment and the qualities desired in the crop.

### Intracanopy lighting

The higher energy efficiency of LEDs ensures that they are much cooler than their MH and HPS equivalents^170^. This allows LEDs to be interspersed within a canopy to ensure greater light distribution throughout a densely planted crop. This has multiple benefits, ranging from greater light use efficiency (and therefore reduced energy consumption^171^), to increase uniformity, quality, and yield of fruit^166,167^. Intracanopy lighting could also be used to control plant architecture; for instance supplemental red light could be used to minimize internode elongation and leaf drop as part of a shade avoidance response. This has particular relevance for leaf crops such as lettuce, where supplemental lighting has been used to limit senescence, thereby enhancing yield^172^.

### Night breaks

Beyond the utilization of supplemental lighting to extend day length and increase the distribution of light in the canopy, short periods of light during the night have been successfully used to manipulate plant development. In short day plants, such as *Chrysanthemum* and *Ipomoea nil*, night breaks using red light can be used to delay flowering^173–175^. Conversely, night breaks can be used to accelerate flowering in long day plants^176^. In tomato, red light night breaks induced a delay in flowering and decreased plant height while also improving tomato fresh weigh shortly after flowering^177^. These differences in flowering and plant morphology are most likely derived from activation of phytochromes (which would otherwise revert to their inactive state in the dark—see the section 'Phytochromes') and it is likely such phenomena will also be observed in other species.

### Post-harvest lighting regimes

Supplemental lighting can also be used after harvesting to prolong shelf-life or to alter the biochemical properties of the crop. For instance, irradiation with white LEDs was sufficient to delay senescence and therefore promote the shelf life of harvested sprouts^178^, whereas irradiation of sweet peppers after harvesting was sufficient to induce color break, thereby enhancing market value^179^. Interestingly, maintenance of circadian rhythms through the utilization of light:dark cycles delays senescence compared to constantly lit conditions, demonstrating the need for further research to more thoroughly understand how complex lighting regimes can be utilized to improve storage of harvested crops^180^.

## Future perspectives

Plants have evolved a sophisticated network of photoreceptors that enable them to perceive and respond to environmental change. As commercial scale installation of LEDs becomes viable, the on-going challenge facing commercial growers will be the optimization of lighting regimes to promote desirable qualities for glasshouse management and crop quality, while also considering the economic costs of LED installation and the specific photoresponsive traits of their crop. Although there are numerous examples of diversification of regulatory pathways, it is reassuring that the photoreceptors and key downstream regulatory modules regulating flowering time, phenylpropanoid biosynthesis, and carotenoid production are conserved. Such conservation demonstrates that it will be possible to utilize the understanding gained from model species to design tailored light regimes optimized for many glasshouse-grown crops, leading to improved yield and quality in the future.
